# Plant-Derived Trimeric CO-26K-Equivalent Epitope Induced Neutralizing Antibodies Against Porcine Epidemic Diarrhea Virus

**DOI:** 10.3389/fimmu.2020.02152

**Published:** 2020-09-16

**Authors:** Thuong Thi Ho, Giang Thu Nguyen, Ngoc Bich Pham, Van Phan Le, Thi Bich Ngoc Trinh, Trang Huyen Vu, Hoang Trong Phan, Udo Conrad, Ha Hoang Chu

**Affiliations:** ^1^Graduate University of Science and Technology, Vietnam Academy of Science and Technology, Ha Noi, Vietnam; ^2^Institute of Biotechnology, Vietnam Academy of Science and Technology, Ha Noi, Vietnam; ^3^Vietnam National University of Agriculture, Ha Noi, Vietnam; ^4^Leibniz Institute of Plant Genetics and Crop Plant Research (IPK), Gatersleben, Germany

**Keywords:** trimeric COE, PEDV, neutralizing antibody, plant-based vaccine, recombinant protein

## Abstract

*Porcine epidemic diarrhea virus* (PEDV) is a causative agent of a highly infectious disease with a high mortality rate, especially in newborn piglets in Asian countries resulting in serious economic loss. The development of a rapid, safe, effective and cost-efficient vaccine is crucial to protect pigs against PEDV infection. The COE antigen is regarded to be a major target for subunit vaccine development against PEDV infection. The naturally assembled COE protein forms a homotrimeric structure. In the present study, we successfully produced a trimeric COE protein as a native structure by fusion with the C-terminal isoleucine zipper trimerization (GCN4pII) motif in *Nicotiana benthamiana*, with a high expression level shown via semi-quantified Western blots. Trimeric COE protein was purified via immobilized metal affinity chromatography (IMAC), and its trimeric structure was successfully demonstrated by a cross-linking reaction, and a native PAGE gel. A crude extract containing the COE trimer was used for evaluating immunogenicity in mice. After 1 and 2 booster immunizations, the crude extract containing trimeric COE elicited elevated PEDV-specific humoral responses, as demonstrated by ELISA and Western blot analyses. Notably, a virus-neutralizing antibody assay indicated that the neutralization activities of sera of mice vaccinated with the crude extract containing COE-GCN4pII were similar to those of mice vaccinated with a commercial vaccine. These results suggest that crude extract containing trimeric COE is a promising plant-based subunit vaccine candidate for PEDV prevention.

## Introduction

Porcine epidemic diarrhea (PED) is a highly infectious disease identified by dehydration, acute watery diarrhea, and a high mortality rate, especially in newborn piglets (1–3). PEDV, the disease causative agent of PED, spreads to several countries in the world, and resulting in serious economic loss to the swineproduction (4–6). The PEDV genome comprises five open reading frames (ORFs) encoding four structural proteins [the spike (S), envelope, membrane and nucleocapsid proteins] and three non-structural proteins [the replicases ORF1a and 1b, and ORF3; (7, 8)]. Among the structural proteins, the S protein locating on the surface of PEDV virion, plays a key role in the attachment of PEDV to host cell receptors (9–11). In addition, the S protein is a target for neutralizing antibody induction because it harbors virus-neutralizing epitopes and is the principle antigenic determinant (8). The S protein is naturally assembled in homotrimeric form with a number of predicted glycosylation sites (12).

The CO-26K-equivalent epitope (COE epitope) is one of the various neutralizing epitopes on the S protein of PEDV which have been recognized (10). COE is the antigen epitope motif that was identified by the monoclonal antibody 2C10 at the C-terminal end of the S protein (13) and the S1D domain (14). The COE protein is regarded as a critical target for the subunit vaccine development against PEDV infection (15). The neutralizing epitope region of COE contains 139 amino acids within the S1 domain extending from amino acid 499–638 (10, 15). COE has been expressed as monomer or pentamer structures in various plants including tobacco, rice, and lettuce (15–19). Mice fed transgenic plants or immunized transgenic rice calli protein extracts containing the monomeric COE protein were found to have both systemic and mucosal immune responses against the COE antigen (15, 16). The immunogenictity tests of pentameric COE have not been tested. To date, the expression of trimeric COE as a native COE structure in plants and immunogenicity of trimeric COE in animals have not been reported.

GCN4, known as the GCN4 leucine zipper, is a yeast transcription factor that is responsible for the reductive reaction of amino acid deficiency (20). GCN4 can be switched from native dimer form to multimeric states by mutations in the a- and d-positions (21). GCN4pII is generated by a core that was formed entirely of beta branched residues. GCN4pII has been used for the successful production of trimeric HA proteins of H5N1 viruses (22, 23), and trimeric S protein of PEDV (12). In which, the GCN4pII was used to trigger trimerization of the proteins of interest, and increase protein stability and solubility.

The development of a rapid, valid, safe, and cost-effective vaccination strategy to protect swine against PEDV is urgently needed, especially in developing Asian countries producing pigs.

Plant-based subunit vaccines have been reported with several advantages including low manufacturing cost, effortlessness of scaling, high stability and long shelf life [for a review, see (24)]. In addition, low profit margins in the industrial vaccine development are provided from plants with beneficial and economical platforms (25). Agro-infiltration methods can offer various advantages in production of substantial amounts of recombinant proteins in short times (a few days) after completing vector construction process, and therefore, this system generally exhibits as a very fast and efficient method to produce subunit vaccines (26).

In this study, we generated a plant vector containing a DNA sequence encoding the COE protein of the PEDV DR13 strain fused GCN4pII as a vector model to investigate the immunogenicity of a plant-based COE antigen in mice compared to that of a commercial vaccine. Interestingly, the neutralization activities of sera of mice vaccinated with the crude extract containing COE trimer were similar to those of mice vaccinated with the commercial vaccine. Our first successful initial results are expected to provide an alternative strategy to generate a plant-based trimeric COE vaccine against PEDV infection for national rapid response.

## Materials and Methods

### Production and Characterization of Plant-Based Recombinant Protein

**Figure 1:**
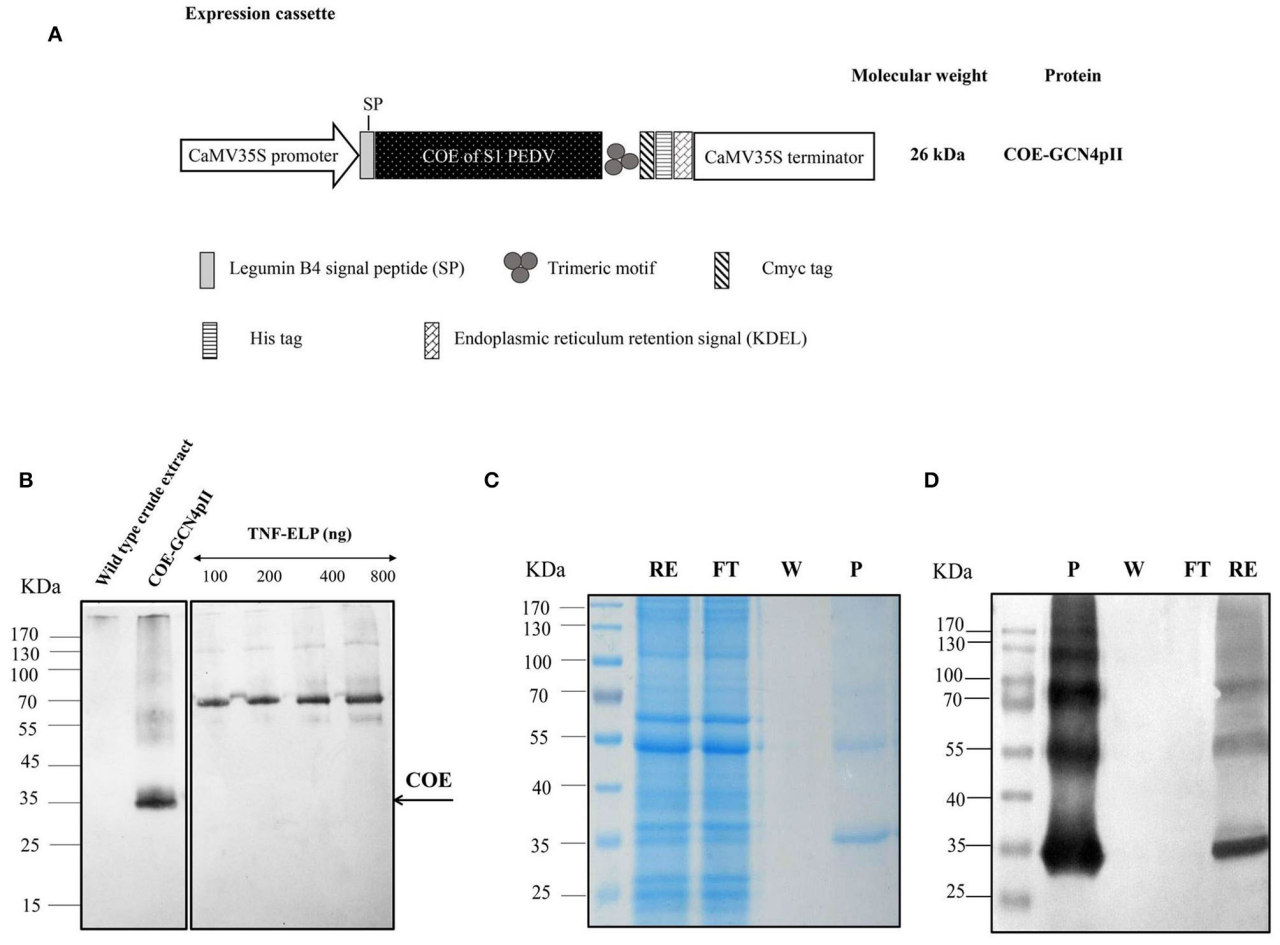
Determination of the expression level and purification of COE-GCN4pII protein in plants. **(A)** The COE sequence encoding the COE protein of PEDV was fused with the C-terminal trimeric motif (GCN4pII) for generating trimeric COE protein. The recombinant protein was fused to a c-myc tag and his-tag for downstream detection by Western blot and protein purification by IMAC, respectively. The legumine B4 signal peptide and the KDEL motif were used to target protein retention in the ER. CaMV35S Pro, cauliflower mosaic virus 35S ubiquitous promoter; CaMV 35S Term, cauliflower mosaic virus 35S terminator; asterisk, the molecular weight of proteins was calculated for unglycosylated monomers. **(B)** A total of 11.25 μg of total soluble proteins in crude extract containing COE-GCN4pII or wild type crude extract and a series of known concentrations of the anti-nanobody-ELP standard (100, 200, 400, and 800 ng) were separated in 4–10% gel polyacrylamide. An anti-cmyc tag antibody was used as a primary antibody. HRP-linked goat anti-mouse IgG was used as a secondary antibody. The COE content in the plant crude extract was determined by comparing the blot signal intensities and those of the standard protein using ImageJ software. **(C)** SDS-PAGE analysis of the purification procedure of COE-GCN4pII from total soluble protein extracts using IMAC; RE, raw extract; FT, flow through; W, wash fraction and P, purified fraction in SDS-PAGE. **(D)** Western blot analysis of the purification procedure of COE-GCN4pII from total soluble protein extracts using IMAC. COE-GCNpII was immunologically detected via an anti-his monoclonal antibody.

The COE nucleotide sequence encoding for amino acid 499–638 of the COE in the S protein of the attenuated PEDV DR13 strain (NCBI accession number JQ023161.1) was synthesized, codon optimized commercially in tobacco (Genebank accenssion number BankIt2361779 Optimized MT761690), and then inserted in a pEZ cloning vector (POCH, Life Science Missouri City, Texas 77489). The COE gene was amplified from the vector, and replaced for the H5 sequence present in pRTRA-CaMV35S-H5-GCN4pII-cmyc-his-KDEL (22) to *via* the *Bam*HI and *Bsp*120I sites. The resulting expression cassette (Figure 1A) was inserted into the expression vector pCB301-Kan (27) via *Hind*III cleavage, then transformed into the *Agrobacterium tumefaciens* [pGV2260 in C58C1; (28)] strain via electroporation at 2.5 kV, 25 μF capacitance, and 400 Ohm resistance.

To express the recombinant protein in *planta*, the agro-infiltration protocol was performed as described by Pham et al. (29), with some modifications. Briefly, bacteria containing the expression vector pCB301-COE-GCN4pII-cmyc-his-KDEL and plant vector including HcPro (23), that was used as a gene silencing suppressor for enhancing the expression levels of targeting proteins in plants (30, 31), were mixed and diluted in an infiltration buffer [10 mM 2-(N-morpholino) ethanesulfonic acid (MES), 10 mM MgSO_4_, pH 5.6]. *N. benthamiana* plants (5 weeks old) were completely infiltrated in an *Agrobacterium* solution, and maintained in a greenhouse. Six days after agro-infiltration, plant leaf samples were collected and stored at −80°C.

Recombinant COE-GCN4pII protein was purified via immobilized metal affinity chromatography *(*IMAC) as described by Pham et al. (29). The oligomeric form of purified COE-GCN4pII protein was determined by a cross-linking reaction that was described by Weldon et al. (32) and a native PAGE.

### Measurement of the COE-GCN4pII Expression Level by SDS-Page and Western Blot

Leaf samples were ground in liquid nitrogen, mixed with PBS buffer (137 mM NaCl, 2.7 mM KCl, 10 mM Na_2_HPO4, 1.8 mM KH_2_PO4, pH 7.4). The crude extract was clarified by centrifugation twice at 13,000 rpm for 30 min at 4°C. The COE-GCN4pII protein in leaf crude extract and a number of known concentrations of anti-TNFα-nanobody-ELP standard protein [100, 200, 400, 800 ng, (33)] were separated in a 4–12% SDS-PAGE gel and transferred to a PVDF membrane (Millipore). The protein expression was determined via Western blot that was performed as described by Pham et al. (29) using monoclonal anti-c-myc antibody. The COE-GCN4pII protein expression level in the leaf crude extract was semi-quantified via Western blotting by comparison of the Western blot signal intensities and those of the anti-TNFα-nanobody-ELP standard protein using ImageJ software.

### Mouse Immunization

The study was approved by the ethical committee of the Institute of Biotechnology, Academic of Science and Technology Vietnam (VAST), Hanoi, Vietnam. The crude extracts after 6 days of the storage were mixed with the Emulsigen®-D adjuvant (MVP Technologies, 4805 G Street, Omaha, NE 68117, USA) with a ratio of 4:1 (v/v), respectively. Three groups of 6–8-week-old female BALB/C mouse (five per group) respectively numbered G1, G2, G3 were subcutaneously vaccinated at days 0, 14 and 28 with 200 μl of Emulsigen®-D adjuvant-formulated crude plant extracts of non-transgenic plants as negative control) or 200 μl of the commercial vaccine against the PEDV DR13 strain (4 × 10^6^ TCID50/dose, CTC Vacc PED, Korea) as positive control or 200 μl of Emulsigen®-D adjuvant-formulated crude plant extracts containing 18.76 μg of COE-GCN4pII protein that was semi-quantified by Western blotting. The bloods of mice were collected at seven days after the second and the third immunization via the retro-orbital sinus. All mouse sera were collected separately by centrifugation. To inactivate the non-specific complement, all mouse sera were incubated at 56°C for 30 min before being stored at −20°C until used.

### Preparation and Purification of PEDV

PEDV propagation and purification were carried out as described by Hofmann et al. (34), with modifications. The Vero E6 cell line (ATCC® CRL-1587TM) was propagated and incubated at 37°C in Dulbecco's Modified Eagle Medium (DMEM) including 10% fetal bovine serum (FBS) and antibiotics (100 μg/mL penicillin/streptomycin). The cells were cultured in a 5% CO_2_ at 37°C. Then, the PEDV-DR13 strain was propagated in Vero cells with 10 μg/mL trypsin treated- tosyl phenylalanyl chloromethyl ketone (TPCK) (Worthington, Lakewood, NJ, USA). After 36 h of cultivation, when all cells showed 100% cytopathogenic effects with morphological changes using cell morphology evaluation by inverted light microscopy, the infective culture fluid was harvested andfreeze-thawed three cycles. Next, cellular debris was pelleted by centrifugation at 10,000 × g for 30 min. The clarified supernatant was then enriched by ultracentrifugation at 30,000 × g. Sucrose density gradient centrifugation (20, 40, 60%) was then used to purify PEDV.

### Detection of PEDV-Specific IgG Antibody Responses by Western Blot

To detect PEDV-specific IgG mouse antibodies, 1 μg of purified PEDV DR13 was loaded onto 3-wells of one SDS-PAGE gel. The virus was separated and transferred to a PVDF membrane. The membranes were blocked with 5% (w/v) fat-free milk in PBS buffer (137 mM NaCl, 2.7 mM KCl, 10 mM Na_2_HPO4, 1.8 mM KH_2_PO4, pH 7.4) for 2 h. To separate the three lanes, the membrane was cut. Next, the single lane was incubated with a mixture of five mouse sera from each group (G1, G2, or G3) with a dilution of 1:200 at room temperature for 2 h, followed by the incubation with goat anti-mouse IgG secondary antibody-conjugated HRP with a dilution of 1:5,000.

### Detection of PEDV-Specific IgG, IgA, IgM Antibody Responses by Indirect ELISA

The indirect ELISA was performed as described by Pham et al. (29), with some modifications. Hundred microliter of purified PEDV DR13 (5 ng/μl) in PBS (100 mM NaCl, 32 mM Na_2_HPO4, 17 mM Na_2_HPO4, pH 7.2) were added in a microtiter plate (ImmunoPlate Maxisorp, Nalgen Nunc International, Roskilde, Denmark) that was then incubated overnight. The plate was blocked, and incubated with 100 μl of each serially diluted mouse serum in 1% (w/v) BSA in PBST at ratios of 1:100, 1:200, 1:400, 1:800, 1:1,600, 1:3,200, 1:6,400, 1:12,800, 1:125,600, 1:51,200, 1:102,400 with three replications, followed by the addition of 100 μl of a 1:10 000 dilution of goat anti-mouse IgG-conjugated HRP (Invitrogen) or 1:2,000 dilution of goat anti-mouse IgA cross-adsorbed secondary antibody-conjugated HRP (Invitrogen) or 1:1,000 dilution of goat anti-mouse IgM secondary antibody-conjugated HRP (Invitrogen) in 1% (w/v) BSA in PBST. An internal control was generated by mixing 5 μl of each serum of all mouse groups, and put in all plates. OD450 signals of internal control were used to normalize the ELISA data. The cut-off value of ELISA was determined as (MEAN + 3.848 × SD) of negative control (35). BSA background was subtracted.

### Virus Neutralization Assay

Two-fold serial dilutions of serum samples after the 2nd immunization were prepared in α-Minimum Essential Medium (MEM) including 1% antibiotic-anti-mycotic solution (Invitrogen, USA). Then, 10^3^ TCID50/0.1 ml of PEDV DR13 was added with an equal volume of diluted serum, and the virus-serum mixture was maintained at 37°C for 1 h. Next, 100 μl of each virus-serum mixture was introduced onto Vero cell monolayers in 96-well plates. The virus-serum mixture was removed after adsorption at 37°C for 1 h. The plates were then washed for 10 min with PBS. Finally, 200 μl of serum-free α-MEM medium containing trypsin were placed into each well and maintained at 37°C for 6 days. For controlling this assay, the virus control, positive serum control, negative serum control and blank control were used. The serum neutralization titres (SN titres) were defined as the highest serum dilution and consequent on inhibition of the cytopathic effect.

### Statistical Analysis

Statistical analyses for the ELISA test and virus neutralization assay were carried out in Sigma Plot software using a *t*-test. The difference between sample data mean was compared and is showed as the X ± standard deviation (SD). *P*-values that were <0.05 were determined to be significantly different.

## Results

### Expression, Purification, and Characterization of the COE-GCN4pII Protein in Plants

The expression of the COE-GCN4pII protein in planta was successful demonstrated via separation of SDS-PAGE under reducing conditions, blotted and detected by Western blot using an anti-c-myc monoclonal antibody (Figure 1B).

The apparent band shown in Figure 1B with COE molecular weight was larger than the expected COE size predicted from the COE polypeptide sequence (26 kDa). One of the possible reasons for the increase in the molecular weight of COE protein is that the N-glycosylation sites located within the COE-S protein of PEDV at amino acids 511 and 533 may influence the electrophoretic behavior during the PAGE separation (16, 17). No COE protein was detected in wild type crude extract. The COE-GCN4pII protein expression level in leaf crude extract was semi-quantified by Western blotting. The COE-GCN4pII protein was quantified with a high expression level of ~4% of the total soluble protein. The amount of plant-produced COE-GCN4pII protein was found to be 234 mg/kg wet weight. Several publications have reported the accumulation of COE in transgenic plants (15, 17, 19); however, the expression level was still lower than that in our report. We demonstrated that the accumulation of COE in tobacco leaves could be significantly improved by codon optimization for plant expression by using a strong expression system, such as agro-infiltration.

**Figure 2:**
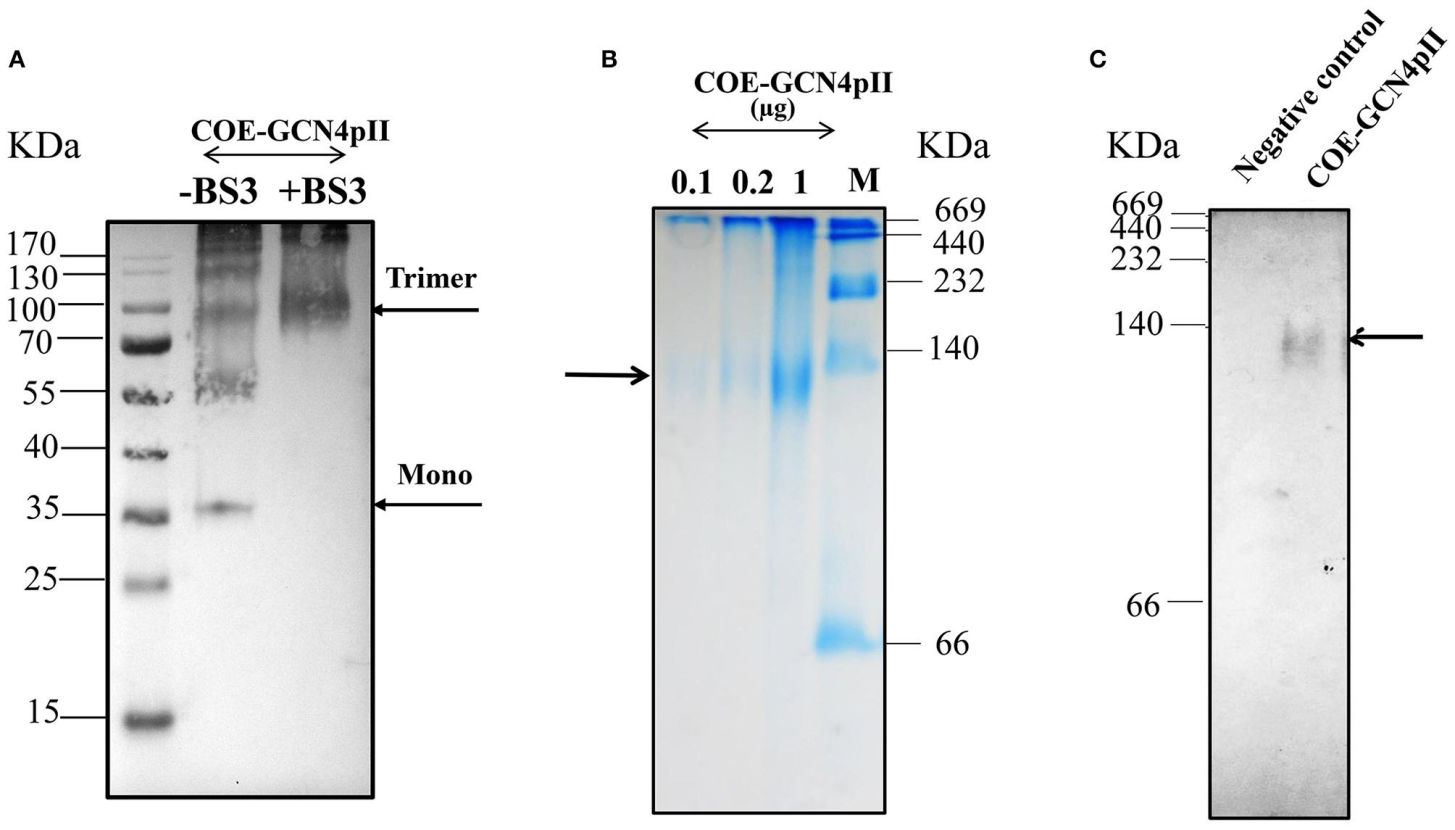
Characterization of oligomeric state of COE protein on SDS-PAGE via cross-linking reaction and native PAGE. **(A)** Detection of the oligomeric states of COE-GCN4pII via a cross-linking reaction; (–) and (+) BS3 indicate the cross-linker BS3 at a 0 and 5 mM final concentration, respectively. The resulting products were separated by SDS-PAGE (4–12% gradient gel) under reducing conditions, blotted and detected by an anti-c-myc monoclonal antibody. **(B)** The COE protein was separated on a 4–15% native polyacrylamide gradient gel under native condition. A native gel was stained in Coomassie staining solution, and washed in destaining solution. M: Amersham high molecular weight calibration kit proteins (GE Healthcare) **(C)**. The native gel was blotted and detected by monoclonal anti-c-myc antibody. Negative control: PBS.

The purification process was validated by collecting samples from each step of the purification procedure to analyse via SDS-PAGE and Western blot using a monoclonal anti-his antibody (Figures 1C,D). These results indicate the enrichment and successful purification of COE-GCN4pII protein from *N. benthamiana* leaves. The oligomeric state of the COE-GCN4pII protein was successfully determined by a cross-linking reaction with BS3 and a separation under native condition by native-PAGE. A band with a molecular weight of approximately 100 kDa corresponding to molecular weight of trimeric COE form was detected (Figures 2A–C). These results revealed that the trimeric COE protein was successfully generated in *planta* by the fusion of COE with GCN4pII motif.

### Strong Immune Responses Induced by the Crude Plant Extract Containing COE-GCN4pII

Since animal vaccine development should minimize downstream processing in pig immunizations, the crude plant extract was chosen for testing immunogenicity in mice. Interestingly, after storing the crude extract containing trimeric COE at 4°C for six days, the COE content was still stable, as revealed by Western blotting (see Supplementary File).

**Figure 3:**
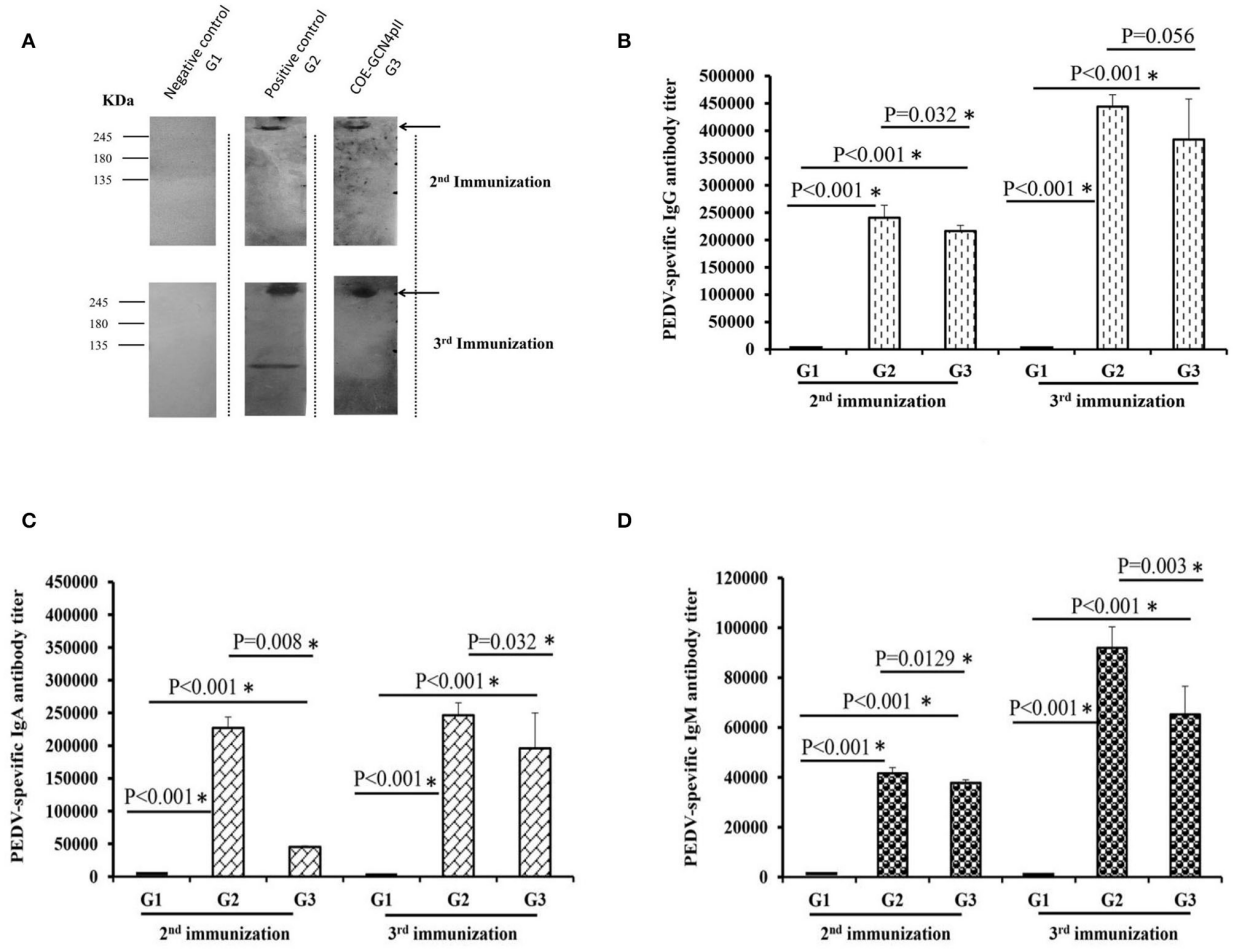
Determination of the levels of PEDV-specific IgG, IgA, and IgM antibody responses via a Western blot and ELISAs. **(A)** Sera from five mice from each group immunized with a negative control (wild-type crude extract, G1) or a positive control (commercial vaccine, G2) or the crude extract containing COE-GCN4pII (G3) were mixed, diluted 200 times and used as a primary antibody for detecting 1 μg of purified PEDV antigen. HRP-linked goat anti-mouse IgG was used as a secondary antibody. In total, 500 ng of purified PEDV per well was coated on a plate. Each serum was measured in triplicate. The sera were serially diluted and analyzed via ELISA. Different levels of PEDV-specific IgG **(B)**, IgA **(C)**, and IgM **(D)** antibodies in each mouse sera group were calculated as the reciprocal of the geometric mean titer of the five mice of each group vaccinated with the negative control (wild-type crude extract, G1) or the positive control (commercial vaccine, G2) or the crude extract containing COE-GCN4pII (G3). End-point titer of IgG, IgA, or IgM antibodies against PEDV of each mouse sera group was compared using the *t*-test (SigmaPlot), and is presented. *P < 0.05 was defined as a statistically significant difference.

The antibody- mediated humoral immune responses from vaccinated mice were first examined against the purified PEDV DR13 strain by Western blot (Figure 3A). Before vaccination, PEDV-specific IgG antibody responses were not detected in mice. The Western blots in Figure 3A showed that there was a band with molecular weight of over 245 kDa detected in mice groups G2 (vaccinated with the commercial vaccine against PEDV) and G3 (vaccinated with crude extract containing COE-GCN4pII) that was larger than the expected size of S protein PEDV (151.38 kDa). The larger band size obtained in Western blot might be explained that might be due to the influence on electrophoretic behavior by the 29 potential N-glycosylation sites locating within the S protein of PEDV during the PAGE separation (36). The results indicate that PEDV-specific IgG antibody responses were induced in mice groups G2 and G3 after the 2nd immunization and the 3rd immunization, and the antibody responses were strongly increased in both mice groups G2 and G3 after the 3rd immunization. In contrast, no PEDV-specific IgG antibody response was detected in mice group G1 vaccinated with crude extracts of non-transgenic plants.

Different levels of PEDV-specific IgG, IgA and IgM antibodies in each mouse sera group were calculated as the reciprocals of the geometric mean titer of the five mice of each group. End-point titer of each mouse sera group was compared by the *t*-test. The results showed that after the 2nd immunization, crude extract containing COE-GCN4pII (G3) elicited elevated levels of IgG, IgA and IgM antibody responses against PEDV (Figures 3B–D), reaching end-point antibody titres of 1:216 178, 1:45 429, 1:37 801, respectively. The levels of IgG, IgA and IgM antibody responses in mice group G3 were strongly enhanced after the 3rd immunization, having end-point antibody titres of 1:383 711, 1:195 985, 1:65 198, respectively. Notably, no statistically significant difference in PEDV-specific IgG antibody responses was obtained between mice group G3 and those in mice group G2, with *P*-values of 0.056 after the third immunization (*P* < 0.05). Therefore, plant crude extract containing the trimeric COE protein had the level of IgG antibodies similar to that of commercial vaccines against the PEDV DR13 strain after the third injection. However, level of PEDV-specific IgA and IgM antibody responses in mice group G3 were lower than those in mice group G2 after the second and the third immunization. Levels of IgG, IgA and IgM antibody responses against PEDV found in the sera of negative control mice group G1 were very poor.

### Neutralizing Antibody Responses Were Induced by the Crude Plant Extract Containing COE-GCN4pII

**Figure 4:**
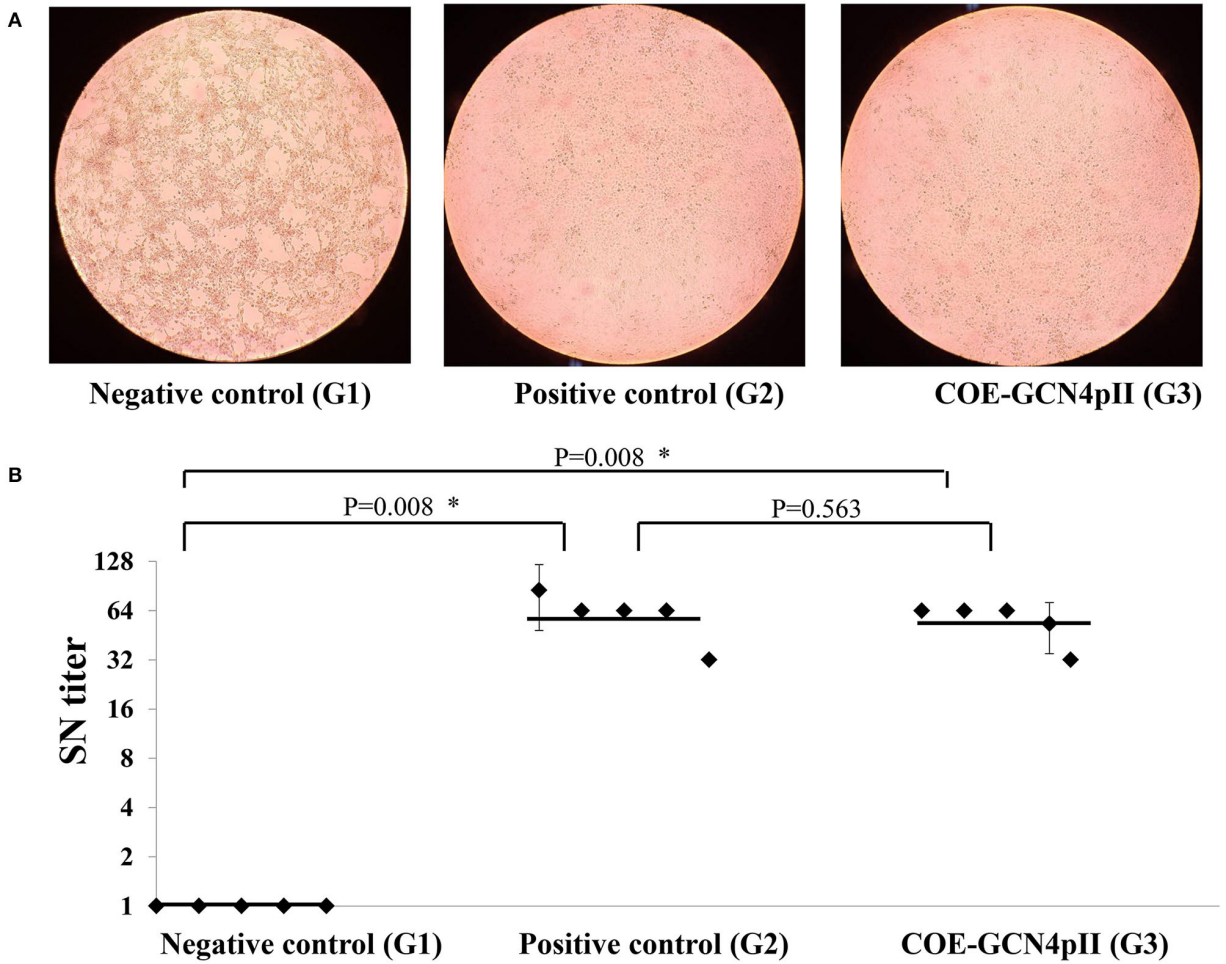
Virus neutralization assay. **(A)** Cytopathic effect caused by wild-type PEDV DR13 virus at the dilution of 1:32 of a single serum from a mouse from the groups vaccinated with the negative control (wild-type plant extract, G1) or the positive control (commercial vaccine, G2) or the crude extract containing COE-GCN4pII (G3) was determined by using a microscope. **(B)** Determination of serum neutralization titer. Serum samples were firstly incubated to inactivate for 30 min at 56°C. Two-fold serial dilutions (1:2, 1:4, 1:8, 1:16, 1:32, 1:64, 1:128, 1:256) were applied. A PEDV DR13 strain (200 μl 10^3^ TCID50/0.1 ml) was mixed with an equal volume of diluted sera. The virus control, positive serum control, blank control, negative serum control, and were used for controlling the assay. The serum neutralizing titres (SN titres) were expressed as the highest serum dilution and consequence on the inhibition of cytopathic effect. Statistical analyses were performed using the *t*-test (SigmaPlot) and are shown. A single dot indicates the SN value of a single mouse serum. The SD is included on a single dot that corresponds to the SN data variation of a single mouse serum with three replications. The bars indicate the average value of the test groups. *P < 0.05 was defined as a statistically significant difference.

The cytopathic effect caused by the wild-type PEDV DR13 virus of all serum samples was determined using a microscope and a representative cytopathic effect result observed under microscope of a single dilution of a serum from each group was presented (Figure 4A). The highest dilutions of sera that caused cytopathic effect inhibition were defined as the serum neutralizing antibody titres. The serum neutralizing antibody (SN) titer of every single mouse serum was presented in each dot (Figure 4B). As expected, cytopathic effect inhibition of sera from the negative control group was observed in a low serum dilution, which produced neutralizing geometric mean titres of this group as low as 1. In contrast, sera of mice vaccinated with the crude extract containing COE-GCN4pII and the commercial PEDV vaccine (the positive control) had the ability to neutralize PEDV with high neutralizing geometric mean titres: 57.6 and 61.86, respectively. These titres were significantly different from those of the negative control group. Notably, the serum neutralization titer in the crude extract containing COE-GCN4pII (G3) was not significantly different compared to that of sera from mice vaccinated with the commercial vaccine, with a *P*-value of 0.187.

## Discussion

In this study, the immunogenicity in mice group G3 vaccinated with the COE trimer in crude extract was compared to those of mice positive control group G2 vaccinated with commercial PEDV vaccine and mice negative control group G1 vaccinated with crude extracts from non-transgenic plants. As expected, Western blot results demonstrated that PEDV-specific IgG antibodies were presented in mice group G3 and mice group G2 after the second and the third immunization. More interestingly, the ELISA analyses illustrated that crude extract containing COE-GCN4pII (G3) elicited strong levels of IgG antibody responses against PEDV, and especially the level of PEDV-specific IgG antibody responses found in mice group G3 was similar to that in mice group G2 after the third immunization. Mucosal immune responses play an important role in the defense of PEDV, and mucosal IgA antibody response was found to correlate with the protection against PEDV infection (37, 38), however IgG antibody-mediated humoral response is also essential to protect the neonatal pig against PEDV infection (37, 39). Early publications showed the important role of IgG in protection of gastrointestinaltract (40, 41). In this study, trimeric COE antigen (G3) was vaccinated in mice via subcutaneous route that is a conventional vaccination route widely used for various human and animal vaccines to elicit IgG antibody-mediated humoral responses. Interestingly, beside PEDV-specific IgG, PEDV-specific IgA and IgM antibodies were presented in mice group G3, however they were lower as compared to those found in mice group G2. Our results are comparable to several recent evidence studies that subcutaneous route can induce both antigen-specific IgG antibody, and antigen-specific IgA and IgM antibodies in sera (42–46). Moreover, Su and his co-workers proposed that under some circumstances (antigen, adjuvant, delivery vehicle) systemic routes may induce systemic immune responses and muscosal immune responses against infectious diseases (46).

In addition, the ability of the neutralizing antibodies induced in mouse sera to neutralize PEDV via binding to COE epitopes related to PEDV neutralization was further determined. After virus neutralization, there was an inhibition generated by neutralizing antibodies to the virus's infection cycle, containing surface binding, fusion, entry, endocytosis, and replication (47). The assessment of PEDV-neutralizing activity illustrated that there was no statistically significant difference between the serum neutralization titres of mice vaccinated with the crude extract containing trimeric COE (G3) and that of mice vaccinated with the commercial vaccine (G2), with a *P*-value of 0.187. Therefore, we concluded that plant crude extract containing the trimeric COE protein had a strong immunogenicity and induced a neutralizing antibody titer similar to that of the commercial vaccine against the attenuated PEDV DR13 strain.

The enhancement of neutralizing antibody responses induced in animals after vaccination is an important requirement for vaccine development due to the powerful correlation of vaccine efficacy with neutralizing antibodies for numerous commercial vaccines (48). The crude extract containing trimeric COE (G3) showed neutralizing activity, with a geometric mean titer of 1:57.6. A high neutralizing antibody titer indicated that mice subcutaneously administered the crude extract containing trimeric COE possessed a strong ability to neutralize PEDV. To increase the immune response, especially mucosal immune responses against PEDV, oral mucosal vaccination with COE but not subcutaneous administration in animals has been previously presented to induce anti-PEDV mucosal immune responses (15, 49). In addition, since PEDV causes mainly intestinal infections, the COE antigen was fused with dendritic cell-targeting peptide (DCpep) and M cell-targeting peptide (Col) for targeting intestinal microfold (M) cells and dendritic cells (DCs) (15, 49, 50). The neutralizing activity mouse sera orally provided genetically engineered Lactobacillus but not plant extracts expressing COE targeting M cells or DCs or both has been previously reported (49, 50). When compared to the publication of Ma and co-workers, the neutralizing antibody titres obtained by oral administration in mice with the recombinant *Lactobacillus casei* strains expressing the PEDV COE antigen on the cell surfaces by fusion with DCpep or Col or both DCpep and Col were 1:24, 1:24, and 1:36, respectively (49), which were lower than the neutralizing antibody titres observed in this study. These results showed that crude extract containing trimeric COE can be a promising vaccine candidate against PEDV infections.

In summary, the trimeric COE protein was successfully produced in plants with high expression levels. Crude extract containing trimeric COE elicited strong humoral immune responses and elevated neutralizing antibody titres against PEDV. In particular, the neutralizing activities of mice vaccinated with the crude extract containing COE-GCN4pII were similar to those of mice vaccinated with the commercial vaccine. These results suggest that crude extract containing trimeric COE might be a potential subunit vaccine antigen against PEDV infection. Further studies will focus on investigating immune efficacy and protection against PEDV in piglets.

## Data Availability Statement

All datasets presented in this study are included in the article/Supplementary Material.

## Ethics Statement

The animal study was reviewed and approved by the principles of the Basel Declaration and recommendations of ARRIVE guidelines, ethical committee of Institute of Biotechnology, Academic of Science and Technology Vietnam (VAST), Hanoi, Vietnam on the use of animals for research.

## Author Contributions

HC, NP, and TH designed the research. TH and GN constructed vectors and performed transient expression. TH purified protein and performed the cross-linking reaction, performed ELISA and Western blotting analyses, and performed the calculations, all data analysis and wrote the manuscript. VL and TT purified the PEDV DR13 strain and carried out the virus-neutralizing antibody assay. UC, HP, NP, TV, and HC revised the manuscript. HC is the corresponding author and holds all the responsibilities related to this manuscript. All authors contributed to the article and approved the submitted version.

## Conflict of Interest

The authors declare that the research was conducted in the absence of any commercial or financial relationships that could be construed as a potential conflict of interest.

## References

[1] Jung K Saif LJ. Porcine epidemic diarrhea virus infection: Etiology, epidemiology, pathogenesis and immunoprophylaxis. Vet J. (2015) 204:134–43. 10.1016/j.tvjl.2015.02.017 25841898 PMC7110711

[2] Stevenson GW Hoang H Schwartz KJ Burrough ER Sun D Madson D. Emergence of Porcine epidemic diarrhea virus in the United States: clinical signs, lesions, and viral genomic sequences. J Vet Diagn Invest. (2013) 25:649–54. 10.1177/1040638713501675 23963154

[3] Chang CY Cheng IC Chang YC Tsai PS Lai SY Huang YL. Identification of neutralizing monoclonal antibodies targeting novel conformational epitopes of the porcine epidemic diarrhoea virus spike protein. Sci Rep. (2019) 9:2529. 10.1038/s41598-019-39844-5 30792462 PMC6385244

[4] Lee S Lee C. Outbreak-related porcine epidemic diarrhea virus strains similar to US Strains, South Korea, 2013. Emerg Infect Dis. (2014) 20:1223–6. 10.3201/eid2007.140294 24960370 PMC4073847

[5] Cima G. PED virus reinfecting US herds. Virus estimated to have killed 7 million-plus pigs. J Am Vet Med Assoc. (2014) 245:166−7. 25115019

[6] Chiou HY Huang YL Deng MC Chang CY Jeng CR Tsai PS. Phylogenetic analysis of the spike (S) gene of the new variants of porcine epidemic diarrhoea virus in Taiwan. Transbound Emerg Dis. (2015) 64:157–66. 10.1111/tbed.12357 25903998

[7] Saif LJ Pensaert MP Sestak K Yeo SG Jung K Coronaviruses. In: Zimmerman JJ, Karriker LA, Ramirez A, Schwartz KJ, Stevenson GW, editors. Diseases of Swine. 10th ed Ames, IW: Wiley-Blackwell (2012). p. 501–24.

[8] Song D Park B. Porcine epidemic diarrhoea virus: a comprehensive review of molecular epidemiology, diagnosis, and vaccines. Virus Genes. (2012) 44:167–75. 10.1007/s11262-012-0713-1 22270324 PMC7089188

[9] Lee SH Yang DK Kim HH Cho IS. Efficacy of inactivated variant porcine epidemic diarrhea virus vaccines in growing pigs. Clin Exp Vaccine Res. (2018) 7:61–9. 10.7774/cevr.2018.7.1.61 29399581 PMC5795046

[10] Chang SH Bae JL Kang TJ Kim J Chung GH Lim CW. Identification of the epitope region capable of inducing neutralizing antibodies against the porcine epidemic diarrhea virus. Mol Cells. (2002) 14:295−9. 12442904

[11] Gallagher TM Buchmeier MJ. Coronavirus spike proteins in viral entry and pathogenesis. Virology. (2001) 279:371–4. 10.1006/viro.2000.0757 11162792 PMC7133764

[12] Walls AC Tortorici MA Bosch BJ Frenz B Rottier PJM DiMaio F. Cryo-electron microscopy structure of a coronavirus spike glycoprotein trimer. Nature. (2016) 531:114–7. 10.1038/nature16988 26855426 PMC5018210

[13] Yang DK Kim HH Lee SH Yoon SS Park JW Cho IS. Isolation and characterization of a new porcine epidemic diarrhea virus variant that occurred in Korea in 2014. J Vet Sci. (2018) 19:71–8. 10.4142/jvs.2018.19.1.71 28693308 PMC5799402

[14] Robert X Gouet P. Deciphering key features in protein structures with the new ENDscript server. Nucleic Acids Res. (2014) 42:W320–4. 10.1093/nar/gku316 24753421 PMC4086106

[15] Huy NX Kim SH Yang MS Kim TG. Immunogenicity of a neutralizing epitope from porcine epidemic diarrhea virus: M cell targeting ligand fusion protein expressed in transgenic rice calli. Plant Cell Rep. (2012) 31:1933–42. 10.1007/s00299-012-1306-0 22736145 PMC7080027

[16] Bae JL Lee JG Kang TJ Jang HS Jang YS Yang MS. Induction of antigen-specific systemic and mucosal immune responses by feeding animals transgenic plants expressing the antigen. Vaccine. (2003) 21:4052–8. 10.1016/S0264-410X(03)00360-8 12922142

[17] Kang TJ Kim YS Jang YS. Expression of the synthetic neutralizing epitope gene of porcine epidemic diarrhea virus in tobacco plants without nicotine. Vaccine. (2005) 23:2294–7. 10.1016/j.vaccine.2005.01.027 15755614

[18] Oszvald M Kang TJ Tomoskozi S Tamas C Tamas L Kim TG. Expression of a synthetic neutralizing epitope of porcine epidemic diarrhea virus fused with synthetic B subunit of Escherichia coli heat labile enterotoxin in rice endosperm. Plant Cell Rep. (2007) 35:215–23. 10.1007/BF02686007 17652785

[19] Huy NX Yang MS Kim TG. Expression of a cholera toxin B subunit-neutralizing epitope of the porcine epidemic diarrhea virus fusion gene in transgenic lettuce (*Lactuca sativa* L). Mol Biotechnol. (2011) 48:201–9. 10.1007/s12033-010-9359-1 21153716

[20] Arndt K Fink GR. GCN4 protein, a positive transcription factor in yeast, binds general control promoters at all 5' TGACTC 3' sequences. Proc Natl Acad Sci USA. (1986) 83:8516–20. 10.1073/pnas.83.22.8516 3464968 PMC386961

[21] Harbury PB Zhang T Kim PS Alber T. A switch between two-, three-, and four-stranded coiled coils in GCN4 leucine zipper mutants. Science. (1993) 262:1401–7. 10.1126/science.8248779 8248779

[22] Phan HT Pohl J Floss DM Rabenstein F Veits J Le BT. ELPylated haemagglutinins produced in tobacco plants induce potentially neutralizing antibodies against H5N1 viruses in mice. Plant Biotechnol J. (2013) 11:582–93. 10.1111/pbi.12049 23398695

[23] Phan HT Ho TT Chu HH Vu TH Gresch U Conrad U. Neutralizing immune responses induced by oligomeric H5N1-hemagglutinins from plants. Vet Res. (2017) 48:53. 10.1186/s13567-017-0458-x 28931425 PMC5607582

[24] Topp E Irwin R McAllister T Lessard M Joensuu JJ Kolotilin I. The case for plantmade veterinary immunotherapeutics. Biotechnol Adv. (2016) 34:597–604. 10.1016/j.biotechadv.2016.02.007 26875776

[25] Shahid N Daniell H. Plant-based oral vaccines against zoonotic and non-zoonotic diseases. Plant Biotechnol J. (2016) 14:2079–99. 10.1111/pbi.12604 27442628 PMC5095797

[26] Rybicki EP. Plant-made vaccines for humans and animals. Plant Biotechnol J. (2010) 8:620–37. 10.1111/j.1467-7652.2010.00507.x 20233333 PMC7167690

[27] Xiang C Han P Lutziger I Wang K Oliver DJ. A mini binary vector series for plant transformation. Plant Mol Biol. (1999) 40:711–7. 10.1023/A:1006201910593 10480394

[28] Deblaere R Bytebier B De Greve H Deboeck F Schell J Van Montagu M. Efficient octopine Ti plasmid-derived vectors for Agrobacterium-mediated gene transfer to plants. Nucleic Acids Res. (1985) 13:4777–88. 10.1093/nar/13.13.4777 4022773 PMC321826

[29] Pham NB Ho TT Nguyen GT Le TT Le NT Chang HC. Nanodiamond enhances immune responses in mice against recombinant HA/H7N9 protein. J Nanobiotechnol. (2017) 15:69. 10.1186/s12951-017-0305-2 28982373 PMC5629800

[30] Conley AJ Joensuu JJ Jevnikar AM Menassa R Brandle JE. Optimization of elastin-like polypeptide fusions for expression and purifcation of recombinant proteins in plants. Biotechnol Bioeng. (2009) 103:562–73. 10.1002/bit.22278 19266472

[31] Sudarshana MR Plesha MA Uratsu SL Falk BW Dandekar AM Huang TK. A chemically inducible cucumber mosaic virus amplicon system for expression of heterologous proteins in plant tissues. Plant Biotechnol J. (2006) 4:551–9. 10.1111/j.1467-7652.2006.00202.x 17309729

[32] Weldon WC Wang BZ Martin MP Koutsonanos DG Skountzou I Compans RW. Enhanced immunogenicity of stabilized trimeric soluble influenza hemagglutinin. PLoS ONE. (2010) 5:e12466. 10.1371/journal.pone.0012466 20824188 PMC2931692

[33] Conrad U Plagmann I Malchow S Sack M Floss DM Kruglov AA. ELPylated anti-human TNF therapeutic single-domain antibodies for prevention of lethal septic shock. Plant Biotechnol J. (2011) 9:22–31. 10.1111/j.1467-7652.2010.00523.x 20444206

[34] Hofmann M Wyler R. Quantitation, biological and physicochemical properties of cell culture-adapted porcine epidemic diarrhea coronavirus (PEDV). Vet Microbiol. (1989) 20:131–42. 10.1016/0378-1135(89)90036-9 2549681 PMC7117183

[35] Frey A Di Canzio J Zurakowski D. A statistically defined endpoint titer determination method for immunoassays. J Immunol Methods. (1998) 221:35–41. 10.1016/S0022-1759(98)00170-7 9894896

[36] Duarte M Laude H. Sequence of the spike protein of the porcine epidemic diarrhoea virus. J Gen Virol. (1994) 75:1195–200. 10.1099/0022-1317-75-5-1195 8176382

[37] de Arriba ML Carvajal A Pozo J Rubio P Mucosal and systemic isotype-specific antibody responses and protection in conventional pigs exposed to virulent or attenuated porcine epidemic diarrhoea virus. Vet Immunol Immunopathol. (2002) 85:85–97. 10.1016/S0165-2427(01)00417-2 11867170

[38] Annamalai T Lin CM Gao X Liu X Lu Z Saif LJ. Cross protective immune responses in nursing piglets infected with a US spike-insertion deletion porcine epidemic diarrhea virus strain and challenged with an original US PEDV strain. Vet Res. (2017) 48:61. 10.1186/s13567-017-0469-7 28985754 PMC6389210

[39] Krishna VD Kim Y Yang M Vannucci F Molitor T Torremorell M. Immune responses to porcine epidemic diarrhea virus (PEDV) in swine and protection against subsequent infection. PLoS ONE. (2020) 15:e0231723. 10.1371/journal.pone.0231723 32343704 PMC7188253

[40] Ward LA Rich ED Besser TE. Role of maternally derived circulating antibodies in protection of neonatal swine against porcine group A rotavirus. J Infect Dis. (1996) 174:276–82. 10.1093/infdis/174.2.276 8699055

[41] Parreno V Hodgins CD de Arriba ML Kang SY Yuan L Ward LA Tô TL Saif LJ. Serum and intestinal isotype antibody responses to Wa human rotavirus in gnotobiotic pigs are modulated by maternal antibodies. J Gen Virol. (1999) 80:1.1417–1.428. 10.1099/0022-1317-80-6-1417 10374959

[42] Hagglund S Hu K Vargmar K Pore L Olofson AS Blodorn K. Bovine respiratory syncytial virus ISCOMs-Immunity, protection and safety in young conventional calves. Vaccine. (2011) 29:8719–30. 10.1016/j.vaccine.2011.07.146 21864616 PMC7115641

[43] Cheng C Pal S Bettahi I Oxford KL Barry PA de la Maza LM. Immunogenicity of a vaccine formulated with the Chlamydia trachomatis serovar F, native major outer membrane protein in a nonhuman primate model. Vaccine. (2011) 29:3456–64. 10.1016/j.vaccine.2011.02.057 21376796 PMC3084512

[44] Hammerschmidt SI Friedrichsen M Boelter J Lyszkiewicz M Kremmer E Pabst O. Retinoic acid induces homing of protective T and B cells to the gut after subcutaneous immunization in mice. J Clin Invest. (2011) 121:3051–61. 10.1172/JCI44262 21737878 PMC3223921

[45] Yang GB Wang Y Babaahmady K Scholler J Rahman D Bunnik E. Immunization with recombinant macaque major histocompatibility complex class I and II and human immunodeficiency virus gp140 inhibits simian-human immunodeficiency virus infection in macaques. J Gen Virol. (2012) 93:1506–18. 10.1099/vir.0.041061-0 22492918

[46] Su F Patel GB Hu S Chen W Induction of mucosal immunity through systemic immunization: phantom or reality?. Human Vacc Immunother. (2016) 12:1070–9. 10.1080/21645515.2015.1114195 PMC4962944 26752023

[47] Klasse PJ Sattentau QJ. Occupancy and mechanism in antibody-mediated neutralization of animal viruses. J Gen Virol. (2002) 83:2091–108. 10.1099/0022-1317-83-9-2091 12185262

[48] Zinkernagel RM. Maternal antibodies, childhood infections, and autoimmune diseases. N Engl J Med. (2001) 345:1331–5. 10.1056/NEJMra012493 11794153

[49] Ma S Wang L Huang X Wang X Chen S Shi W. Oral recombinant Lactobacillus vaccine targeting the intestinal microfold cells and dendritic cells for delivering the core neutralizing epitope of porcine epidemic diarrhea virus. Microb Cell Fact. (2018) 17:20. 10.1186/s12934-018-0861-7 29426335 PMC5807822

[50] Wang X Wang L Huang X Ma S Yu M Shi W. Oral delivery of probiotics expressing dendritic cell-targeting peptide fused with porcine epidemic diarrhea virus COE antigen: a promising vaccine strategy against PEDV. Viruses. (2017) 9:312. 10.3390/v9110312 29068402 PMC5707519

