# Supplementary figures and images for: Plant-Derived Trimeric CO-26K-Equivalent Epitope Induced Neutralizing Antibodies Against Porcine Epidemic Diarrhea Virus
Source: Front Immunol. 2020 Sep 16;11:2152. doi: 10.3389/fimmu.2020.02152 (PMC7524870; doi:10.3389/fimmu.2020.02152)

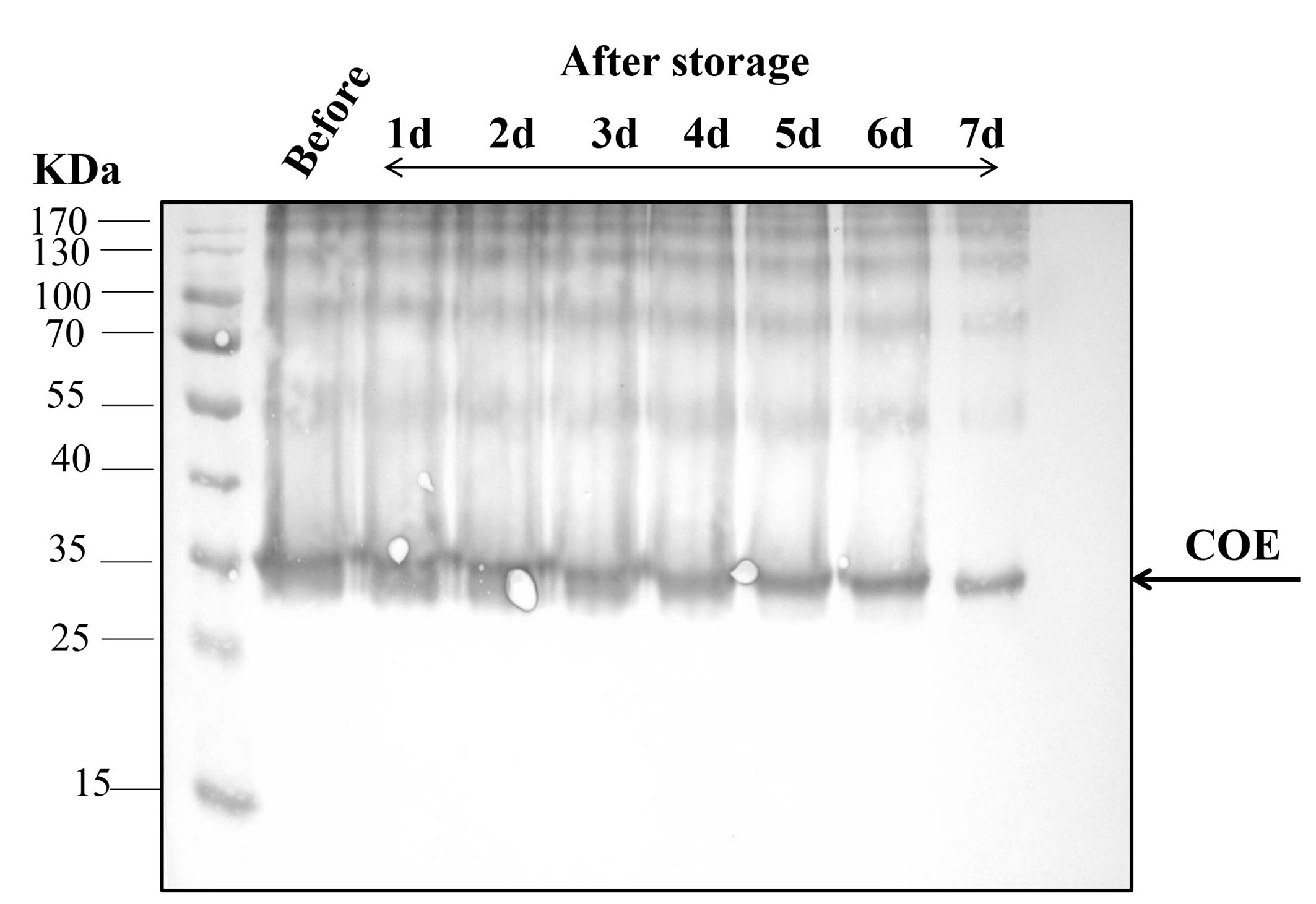

Supplement: Supplementary file 1 [file Image_1.JPEG]
